# Provings and Clinical Observations with High Potencies

**Published:** 1894-02

**Authors:** Malcolm Macfarlan

**Affiliations:** Philadelphia, Pa.


### PROVINGS AND CLINICAL OBSERVATIONS WITH HIGH POTENCIES.

MALCOLM MACFARLAN, M. D., PHILADELPHIA, PA.

### PSORIN.$^{42M}$.

Pain through the right groin when walking ; soreness in glands.

### RANUNCULUS-ACRIS$^{10M}$.

Extremities hurt her very much from knees downward, as if sore ; soreness mostly in the skin.

### RHUS-TOX.$^{105M}$.

Soreness in calves. Severe, intolerable, and uncontrollable itching of feet and legs at night. Slept very little for three days and nights because of itching in the feet. Cured chilblains. Constantly rubbing one foot against the other to relieve itching and pain.

### SAP-SODA$^{2C}$.

Under surface of both heels ache, right worse than left.

### STRONTIA-CARB.$^{5C}$.

Violent pain from her knee to the end of her toes, like sharp rheumatic twinges.

### SULPHUR$^{CM}$.

Great weakness in the legs ; can hardly walk ; an early symptom in the proving.

Produced complete paralysis of the lower extremities. This condition appearing after taking the medicine for two weeks, twenty times a day. One prover only.

### SAMBUCUS$^{45M}$.

Feels very tired on the least exertion; legs feel very tired.

### SASSAFRAS$^{5C}$.

Pain in both tubers ischii and intense pain in both hip joints.

### SECALE$^{95M}$.

Highly curative in the varicose ulcers of the legs in old people.

Gives great relief to those suffering from the ill-effects of milk leg or enlarged and obstructed veins.

### TROMBIDIUM$^{2C}$.

Seems as if the three smaller toes on the left foot were twisted, they hurt her so.

### VERBASCUM$^{45M}$.

Severe pain, as if the parts had been pierced with a knife through the inside of the left ankle joint.

Sensation of great stiffness in ankle joints; left mostly affected.

### ZINC$^{5C}$.

Highly curative in certain old ulcers of the leg, not varicose.

### TIME.

### ARSENIC$^{6M}$.

Cannot sleep after 3 A. M. Wakeful and anxious, highly characteristic.

Fever and sweat occurring every afternoon, or toward night.

Restless at night.

Vivid, frightening dreams.

APOCYN.$^{6M}$.

Restless at night; mind active; thoughts fly rapidly from one thing to another.

AILANTHUS$^{45M}$.

Itching always worse at night.

CIMICIFUGA-RAC.$^{95M}$.

Fever every afternoon, between twelve and four. This noticed in a number of provers.

CUPRUM-ACET.$^{45M}$.

Worse in afternoon or toward evening, and better in forenoon.

SECALE$^{95M}$.

Always worse in afternoon about four o'clock.

TEREBINTH$^{17M}$.

Gets up at night to pass water. Urinary symptoms greatly aggravated at night.

### DISEASES.

ARSENIC$^{6M}$.

This and other high preparations have frequently cured chills and fever where Quinine and other preparations had been given without effect—most useful in old cases where Quinine did no good.

BELLAD.$^{101M}$.

Caused a rash like scarlet fever, with very sore throat—very frequently verified this symptom with high potencies.

BARYTA$^{CM}$.

Highly curative in chronic enlargement of the lymphatic glands of the neck.

BELLAD.$^{101M}$.

This remedy is the most generally useful one in chronic inflammation of the eyelids with intolerance of light and pain—Arsenic high is second in importance.

### DIGIT.$^{CM}$.

Repeatedly relieved the strangury and painful erection of gonorrhœa with this remedy.

### GRAPHITES$^{2X}$.

Highly curative in ophthalmia tarsi.

### KREOSOTE$^{CM}$.

From repeated trials I believe it to be more curative in children's eruptive fevers than Sulph.; most useful in debility, with vomiting. I do not know of a more generally useful remedy in stomach troubles of nearly every kind.

### LACHESIS$^{CM}$.

Wonderfully curative in delirium tremens of drunkards. Has frequently apparently cured membranous croup.

Diphtheritic sore throat and malignant scarlet fever; rapidly helps cases where breath is very offensive; glands of neck swollen.

Suffocative attacks; has to loosen everything around the neck. The very first symptom noticed in proving this remedy is referred to the throat and is a feeling of tightness or suffocation; can't breathe well.

### LITHIUM-CARB.$^{5C}$.

Very curative in moist eczema with much itching; syphilitic ulcerations.

### LOBELIA$^{45M}$.

Asthma; the oppression is felt at the larynx mostly; sick stomach during pregnancy, with ptyalism.

### LYC.$^{45}$.

*Gall-stone colic* speedily cured and the acute pain quickly relieved.

### IODIDE-POTASSIUM.

Rheumatic pains in all the joints; eruption at first like flea-bites; later on looks like pimples.

### MERC-PROT-JOD$^{2C}$.

Changes for the better a Hunterian chancre in a very few days.

### MERC-SOL.$^{101M}$.

*Jaundice* with large amount of bile in urine, coming on suddenly in two days. Was cured and urine natural in twenty-four hours after a single dose.

### NATRUM-MUR.$^{CM}$.

One dose, high, cured a long existing case of chills and fever where Quinine had been given with no effect. Repeatedly verified this observation.

### PETROLEUM$^{CM}$.

Useful in cases of fistula ani; those who have fistula are disposed to eczema.

*Eczema* cured in all its forms is favorably affected by this remedy.

### PHYTOLACCA$^{2C}$.

Boy with anterior and lateral curvature of the spine complained of severe pains across buttocks; relieved in a short time; was enabled to walk across the floor for the first time in four years.

Psorinum$^{42M}$, Kreosote$^{CM}$, Causticum$^{30M}$, and Silicea have cured many cases of otorrhœa lasting from months to years.

### RHUS$^{105M}$.

Cured several cases with every symptom of consumption.

Has helped several very bad cases for a while, especially as to chest pains.

Wonderfully curative in contagious scrofulous ophthalmia of children.

Ecthyma usually cured by Rhus.

Often cured tinea tonsurans in children.

Good result from Rhus in curing the convulsive muscular movements of typhoid fever.

Rhus-tox. is *the* remedy in many cases of scarlet fever. See the provings under that head, skin symptoms, vomiting, etc.

### SULPHUR$^{2C}$.

*Epilepsy* cured several times with Sulphur. Excellent remedy for one who has been overcome with the sun's rays.

Boy, æt. two and a half years, cured in five weeks of complete paralysis, lower extremities.

Chronic granular inflamed lids, with itching, burning, and profuse lachrymation; great photophobia cured quickly.

### SECALE$^{95M}$.

Many cases of varicose ulcers; veins diminish in size.

### SILICEA$^{CM}$.

Cures onychia, run-arounds, inverted nails; often verified.

### SULPH.$^{CM}$.

Followed by Phos.$^{CM}$, curative in *asthenopia*, young woman, eighteen; severe case, existing one and a half years.

### TART-EMET.$^{5M}$.

Highly curative in dyspepsia or sick stomach, with soreness throughout the chest.

### TRITICUM$^{5}$.

Cold in the head with coughing, sneezing; nose discharges freely.

### VARIOLIN$^{45M}$.

Has proven wonderfully curative in *small-pox;* modifies it. Frequently verified this in the epidemic of 1873.

### GLAUCOMA.

He was entirely blind in the left eye, which had been lost in a similar manner (glaucoma); now almost crazy with pain in the other; dimness of sight, etc.; the right eye was cured by Sulph., Bell.$^{101M}$, Mezereum$^{103}$.

*Gonorrhœa,* Cop., Tereb., Dig., Petros., Sulph., Sep., Canth., Thuja.

